# Association between Single Nucleotide Polymorphisms in *XRCC3* and Radiation-Induced Adverse Effects on Normal Tissue: A Meta-Analysis

**DOI:** 10.1371/journal.pone.0130388

**Published:** 2015-06-19

**Authors:** Yu-Zhe Song, Fu-Jun Han, Min Liu, Cheng-Cheng Xia, Wei-Yan Shi, Li-Hua Dong

**Affiliations:** 1 Department of Radiation Oncology, the First Hospital of Jilin University, Changchun, Jilin, China; 2 Cancer Center, the First Hospital of Jilin University, Changchun, Jilin, China; University Medical Center Hamburg-Eppendorf, GERMANY

## Abstract

The X-ray repair cross-complementing group 3 (*XRCC3*) protein plays an important role in the repair of DNA double-strand breaks. The relationship between *XRCC3* polymorphisms and the risk of radiation-induced adverse effects on normal tissue remains inconclusive. Thus, we performed a meta-analysis to elucidate the association between *XRCC3* polymorphisms and radiation-induced adverse effects on normal tissue. All eligible studies up to December 2014 were identified through a search of the PubMed, Embase and Web of Science databases. Seventeen studies involving 656 cases and 2193 controls were ultimately included in this meta-analysis. The pooled odds ratios (ORs) with corresponding 95% confidence intervals (CIs) were calculated to evaluate the association between *XRCC3* polymorphisms and the risk of radiation-induced normal tissue adverse effects. We found that the *XRCC3* p.Thr241Met (rs861539) polymorphism was significantly associated with early adverse effects induced by radiotherapy (OR = 1.99, 95%CI: 1.31–3.01, *P* = 0.001). A positive association lacking statistical significance with late adverse effects was also identified (OR = 1.28, 95%CI: 0.97–1.68, P = 0.08). In addition, the rs861539 polymorphism was significantly correlated with a higher risk of adverse effects induced by head and neck area irradiation (OR = 2.41, 95%CI: 1.49–3.89, *p* = 0.0003) and breast irradiation (OR = 1.41, 95%CI: 1.02–1.95, *p* = 0.04), whereas the correlation was not significant for lung irradiation or pelvic irradiation. Furthermore, *XRCC3* rs1799794 polymorphism may have a protective effect against late adverse effects induced by radiotherapy (OR = 0.47, 95%CI: 0.26–0.86, *P* = 0.01). Well-designed large-scale clinical studies are required to further validate our results.

## Introduction

Radiotherapy is an important and commonly used modality in cancer treatment, but normal tissues both in the vicinity of the target area and the pathway of the radiation beam are inevitably irradiated, which may result in a spectrum of normal tissue adverse effects [1]. The prescribed dose of radiotherapy in most malignant diseases is restricted by the tolerance of normal tissue to radiation [2]. However, patients exhibit large variability in normal tissue toxicity even to the same treatment schedule [3]. Some patients display hyper-sensitivity to standard radiotherapy, while typically sensitive patients can receive higher doses of radiotherapy improving the likelihood of a cure for malignant tumors [4]. If the individual risk of adverse effects can be predicted prior to radiotherapy, it would be of great benefit to the personalization and optimization of treatment strategies [5–6].

In recent years, accumulating evidence has supported the hypothesis that the risk of radiotoxicity correlates with genetic susceptibility [5,7]. Single nucleotide polymorphisms (SNPs) account for most known genetic variation [6,8]. By altering the amino acid composition of the encoded proteins, SNPs in DNA repair genes may alter protein function and an individual’s capacity for the repair of damaged DNA [9–11].

DNA is widely considered the main target of radiotherapy, which causes cell death by inducing both single-strand breaks (SSBs) and double-strand breaks (DSBs) [12]. DSBs are potent inducers of mutations and cell death and occur frequently following irradiation [13–15]. X-ray repair cross-complementing group 3 (*XRCC3*), a member of the *RAD51*-related protein family [16], participates in homologous recombination (HR) repair for DNA DSBs [17] and plays an important role in maintaining chromosome stability and DNA damage repair [18–19]. Several trials have been conducted based on the hypothesis that SNPs in genes involved in DNA repair may interfere with an individual’s DNA repair capacity and thus further influence the occurrence of radiation-induced adverse effects [20–22]. Previous studies on the association between *XRCC3* polymorphisms and radiation-induced adverse effects have reported inconclusive results. Thus, we reviewed this controversial evidence and performed a meta-analysis to evaluate the association between *XRCC3* polymorphisms and the risk of radiation-induced adverse effects.

## Materials and Methods

### Search strategy and inclusion criteria

Two investigators (Y.Z. Song and C.C. Xia) independently searched the PubMed, Embase and Web of Science databases using the terms “polymorphism” or “SNP,” “*XRCC3*” or “X-ray repair cross-complementing group 3,” “radiotherapy” or “radiation,” and “injury” or “toxicity” or “adverse effect” or “complication.” Studies satisfying the following criteria were eligible for inclusion: (1) case-control study or cohort study; (2) evaluated the effect of SNPs in *XRCC3* on radiation-induced adverse effects on normal tissue; (3) adequate information provided to calculate the odds ratio (OR) and the corresponding 95% confidence interval (95%CI). There were no limitations on the language of publication. To avoid exaggerating the effects of certain studies, the patient cohorts of the included studies were verified to ensure that only one appropriate comparison from each cohort was included for the calculation of the pooled statistic.

### Data extraction

Two investigators (Y.Z. Song and W.Y. Shi) independently extracted data from each included study. Disagreements were resolved by discussion among all investigators. The following data were extracted: first author, year of publication, ethnicity, cancer type, normal tissue toxicity, subtype of SNP in *XRCC3*, and numbers of cases and controls who possessed the major allele homozygote, heterozygote and minor allele homozygote genotypes.

### Statistical methods

The pooled OR and 95%CI were calculated to assess the association strength between *XRCC3* polymorphisms and the risk of adverse effects induced by radiotherapy under a dominance model (minor allele homozygote /heterozygote vs. major allele homozygote). The heterogeneity between studies was assessed with the chi-squared based Q-test and *I* ^2^ statistics [23–24]. When the chi-squared *P* was <0.10 or the *I* ^2^ statistic was ≥50%, the heterogeneity was considered statistically significant, and a random-effects model (DerSimonian-Laird method) was used [25]; otherwise, a fixed-effects model (Mantel-Haenszel method) was accepted [26]. If more than 10 studies were included for one SNP, subgroup analysis was conducted by adverse effect and irradiation area. Sensitivity analysis was performed to confirm the stability and reliability of the pooled results by excluding each study individually and recalculating the pooled ORs and 95%CIs. If more than 10 studies were included in this meta-analysis, publication bias was evaluated via Begg’s funnel plot and Egger’s test [27–28]. If publication bias existed, the “trim and fill” method was used to estimate the number of missing studies and to adjust the pooled result [29]. A two-sided *P* <0.05 was considered significant for all of the analyses except the heterogeneity tests. Statistical analyses were performed using Review Manager (Rev-Man version 5.0, provided by the Cochrane Collaboration, Oxford, England) and STATA (Version 12.0, StataCorp LP, College Station, TX, USA).

## Results

### Eligible studies

**Fig 1:**
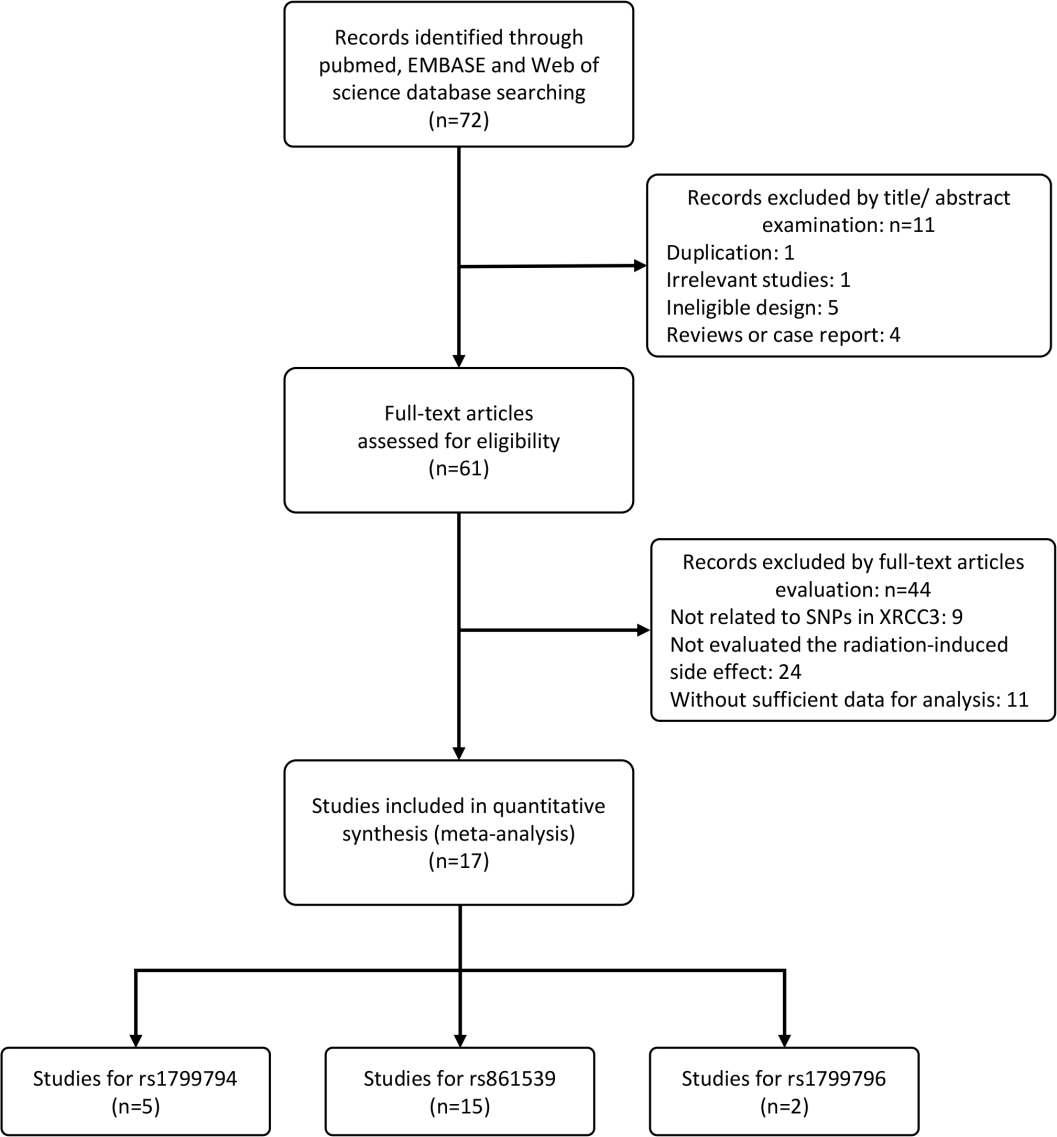
Flow diagram of the literature review process.

A flow diagram summarizing the literature review process and reasons for exclusion is presented in Fig 1. A total of 17 studies involving 2849 patients were eventually included in this meta-analysis. The baseline characteristics of the 17 studies are presented in Table 1. All protocols of the 17 included studies were approved by the relevant ethics committee. The studies were published from 2005 to 2014, and the sample sizes ranged from 34 to 698. The cancer categories included head and neck cancer (five studies), breast cancer (five studies), prostatic carcinoma (two studies), non-small cell lung cancer (two studies), bladder cancer (one study) and gynecologic cancer (one study). In addition, one study included miscellaneous cancers (mainly breast cancer and head and neck cancer). Three subtypes of SNPs in *XRCC3* were included in this meta-analysis. Fifteen studies were identified for rs861539, five studies for rs1799794 and two studies for rs1799796. For rs861539, six studies evaluated the early adverse effects induced by radiotherapy, while nine studies focused on late adverse effects.

**Table 1:** Baseline Characteristics of the Eligible Studies.

Author, Year	Country	Ethnicity	Disease	SNP	Adverse Effect	Assessment Criteria	Sample Size (N)	Cases/N	Study Design	EBRT Dose, Gy	Chemotherapy Involved
Alsbeih 2010[30]	Saudi Arabia	Asian	HNC	rs861539	Late effect: fibrosis	RTOG/EORTC≥G2	60	50%	Case-control	66–70	Yes
Azria 2008[7]	France	Caucasian	Mixed^a^	rs861539	Late effect: fibrosis	CTCAE v3.0≥G3	34	47.06%	Case-control	NA	Yes
Burri 2008[31]	USA	Mixed	Prostate cancer	rs861539	Late effect: rectal bleeding, urinary morbidity, erectile dysfunction	RTOG/EORTC≥G1	135	9.36%	Cohort	45 Gy and/ OR brachytherapy	NA
Chang-Claude 2009[32]	Germany	Caucasian	Breast cancer	rs861539	Late effect: telangiectasia	RTOG/EORTC≥G2	401	31.67%	Cohort	55–70	No
Cheuk 2014[33]	China	Asian	HNC	rs861539, rs1799794	Late effect: fibrosis	RTOG≥G1	120	24.17%	Cohort	66–76	Yes
De Ruyck 2005[8]	Belgium	Caucasian	Gynecologic cancer^b^	rs861539, rs1799794, rs1799796	Late effect: side effect in the pelvic area	CTCAE v3.0≥G2	62	35.48%	Cohort	45–66 and/or brachytherapy	Yes
Fachal 2012[34]	Spain	Caucasian	Prostate cancer	rs1799794	Early effect: gastrointestinal morbidity, genitourinary morbidity	CTCAE v3.0≥G2	698	4.87%	Cohort	70–76	NA
Falvo 2011[35]	Italy	Caucasian	Breast cancer	rs861539	Early effect: acute skin toxicity	CTCAE v3.0≥G1	57	33.33%	Cohort	18–21	Yes
Flavo 2012[36]	Italy	Caucasian	Breast cancer	rs1799794	Late effect: fibrosis or fat necrosis	CTCAE v3.0≥G2	57	45.61%	Cohort	18–21	Yes
Mangoni 2011[37]	Italy	Caucasian	Breast cancer	rs861539	Early effect: acute skin toxicity	CTCAE v2.0≥G2c ^c^	61	11.48%	Cohort	50–62.8	Yes
Popanda 2006[38]	Germany	Caucasian	Breast cancer	rs861539	Early effect: acute skin toxicity	CTCAE v2.0≥G2	444	17.12%	Cohort	49.2–58.8	NA
Pretasi 2011[39]	Italy	Caucasian	HNC	rs861539	Early effect: mucositis	CTCAE v3.0≥G2	101	67.33%	Cohort	54–70	Yes
Sakano 2010[40]	Japan	Asian	Bladder cancer	rs861539	Early effect: gastrointestinal morbidity	CTCAE v3.0≥G2	94	9.57%	Cohort	30.0–60.4	Yes
Tucker 2013[41]	USA	Caucasian	NSCLC	rs861539	Late effect: radiation pneumonitis	CTCAE v3.0≥G3	141	19.86%	Cohort	50.4–72	Yes
Werbrouck 2009[42]	Belgium	Caucasian	HNC	rs861539, rs1799794, rs1799796	Early effect: mucositis, dysphagia	CTCAE v3.0≥G3	85	32.94%	Cohort	66–69	Yes
Yin 2011[43]	USA	Caucasian	NSCLC	rs861539	Late effect: radiation pneumonitis	CTCAE v3.0≥G1	196	69.90%	Cohort	60–70 (majority)	Yes
Zou 2014[11]	China	Asian	HNC	rs861539	Late effect: xerostomia	≥G 1 ^d^	103	41.75%	Cohort	70	Yes

Abbreviations: HNC = head and neck cancer, RTOG = the radiation therapy oncology group, EORTC = European Organization for Research and Treatment of Cancer, EBRT = external beam radiation therapy, CTCAE = Common Terminology Criteria for Adverse Events. NA: not available

^a^ Breast cancer, HNC and meningioma;

^b^ Cervical cancer and endometrial cancer;

^c^ method based on CTCAE, in which G2c was defined as at least one moist desquamation or interruption of radiotherapy due to toxicity.

^d^ method developed by University of Michigan;

### Meta-analysis results

#### Early effect

**Fig 2:**
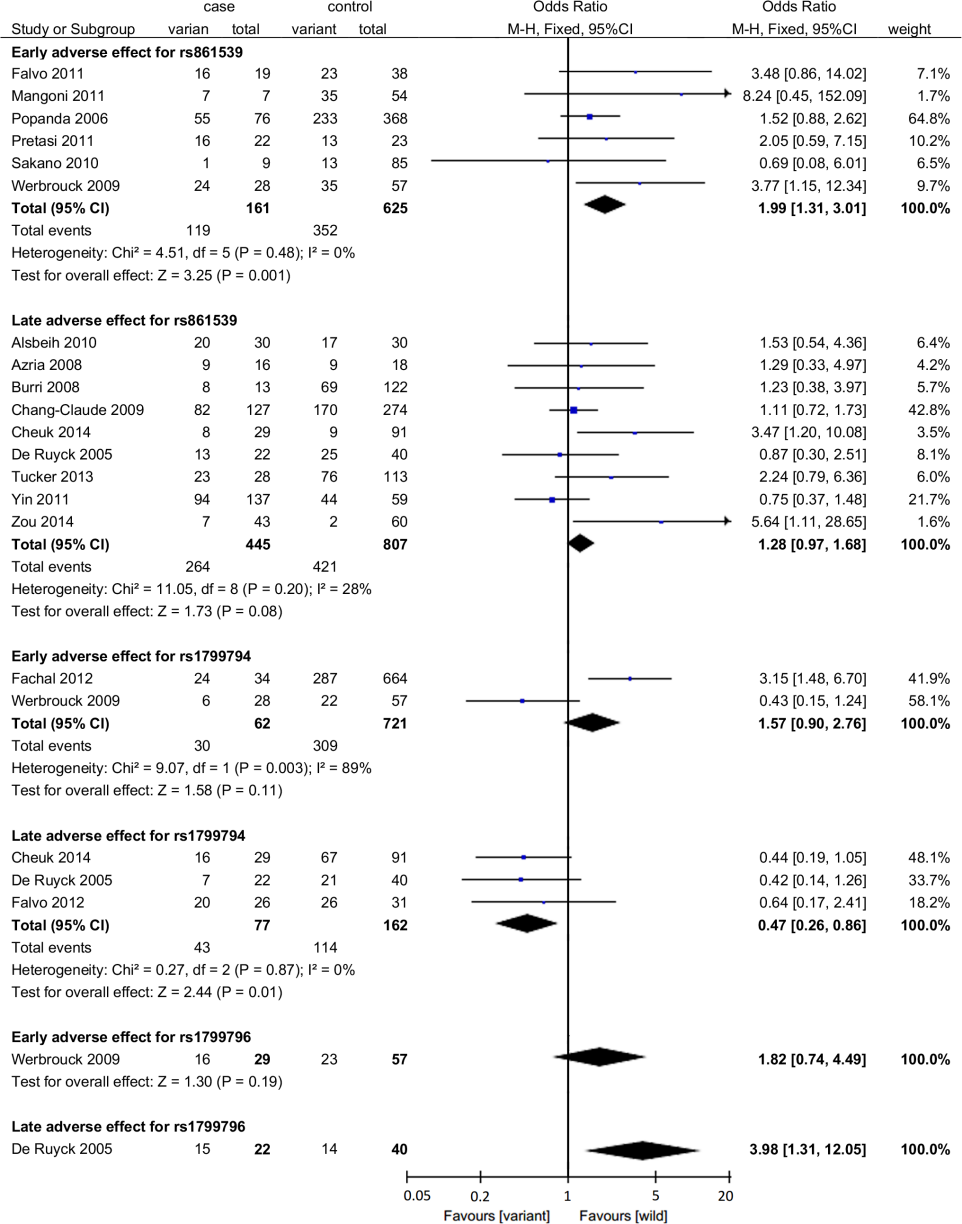
Forrest plot for the association between SNPs in *XRCC3* and radiation-induced adverse effects. A fixed-effects model was used. The square with the corresponding horizontal line represents the OR and 95%CI of each study. The area of the square reflects the weight of the study. The diamond represents the pooled OR and 95%CI.

**Fig 3:**
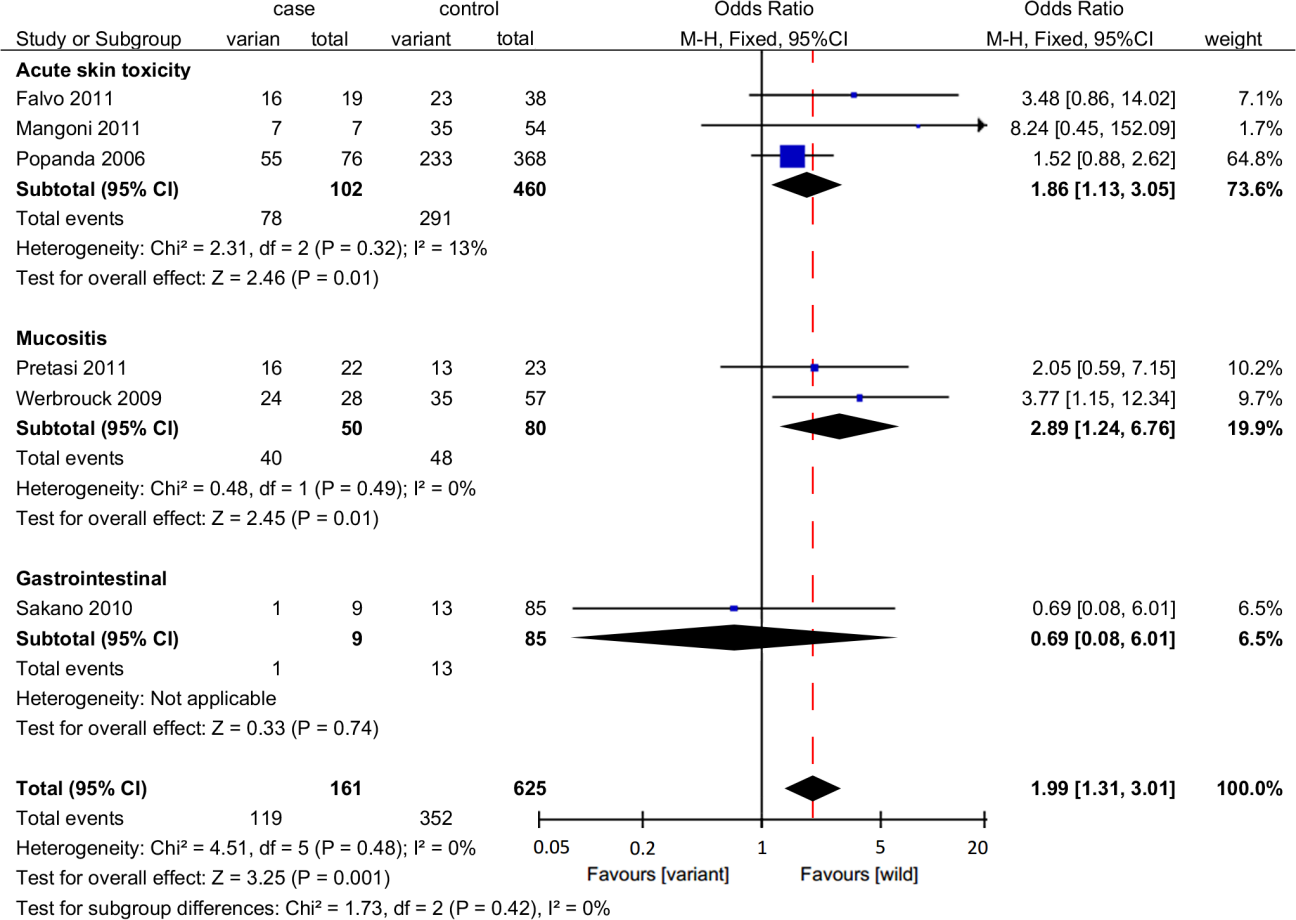
Forrest plot for the association between rs861539 and radiation-induced early adverse effects by specific adverse effect. A fixed-effects model was used. The square with the corresponding horizontal line represents the OR and 95%CI of each study. The area of the square reflects the weight of the study. The diamond represents the pooled OR and 95%CI.

A statistically significant association was identified between rs861539 and early adverse effects induced by radiotherapy (OR = 1.99, 95%CI: 1.31–3.01, *P* = 0.001) (Fig 2). Subgroup analysis was conducted by specific adverse effect, and the results indicated that rs861539 significantly correlated with acute skin toxicity (OR = 1.86, 95%CI: 1.13–3.05, *P* = 0.01) and mucositis (OR = 2.89, 95%CI: 1.24–6.76, *P* = 0.01) (Fig 3). For rs1799794 and rs1799796, the number of identified studies was relatively small, and the statistical associations were not significant (rs1799794: two studies, OR = 1.57, 95%CI: 0.90–2.76, *P* = 0.11; rs1799796: one study, OR = 1.82, 95%CI: 0.74–4.49, *P* = 0.19) (Fig 2).

#### Late effect

**Fig 4:**
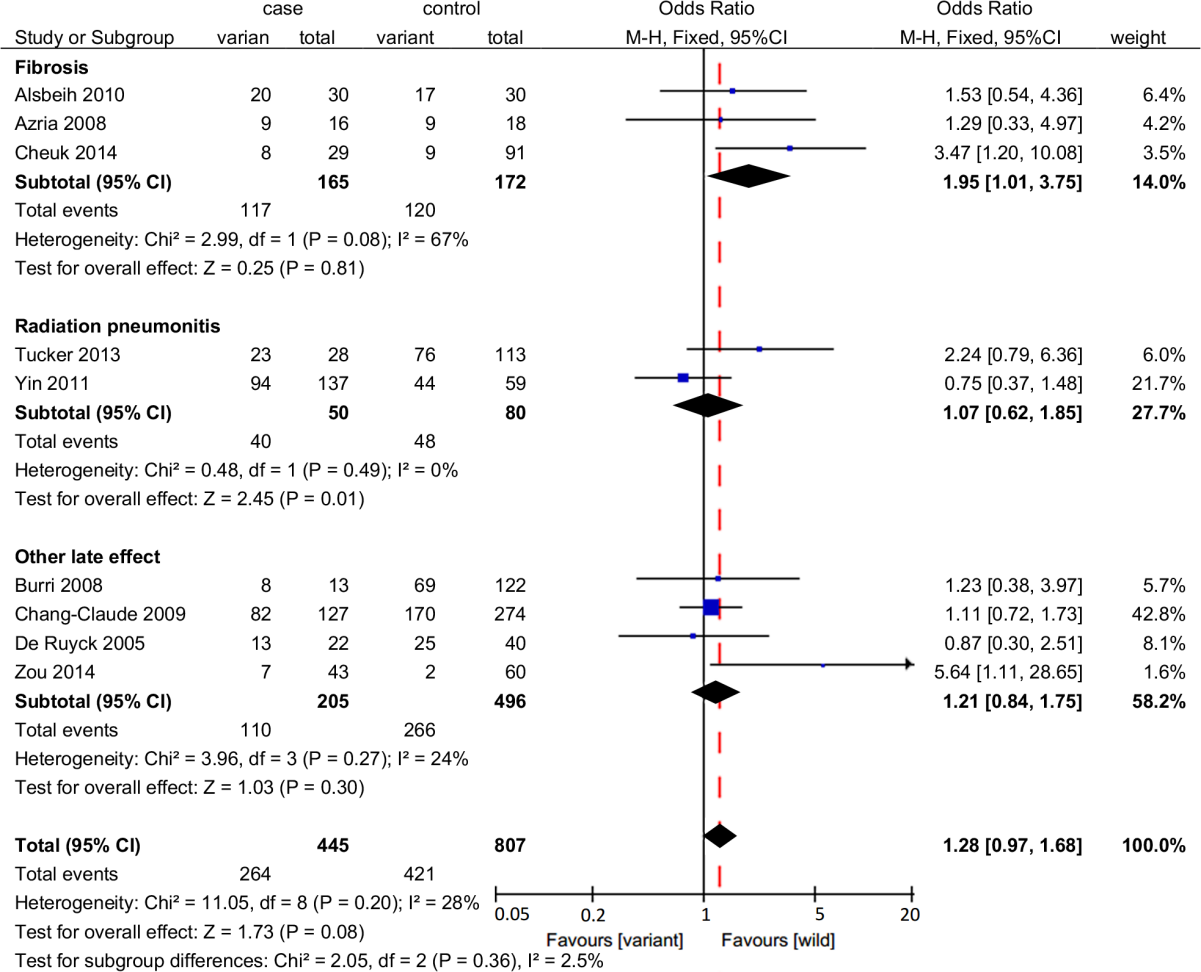
Forrest plot for the association between rs861539 and radiation-induced late adverse effects by specific adverse effect. A fixed-effects model was used. The square with the corresponding horizontal line represents the OR and 95%CI of each study. The area of the square reflects the weight of the study. The diamond represents the pooled OR and 95%CI.

A positive association lacking statistical significance was identified between rs861539 and late adverse effects (OR = 1.28, 95%CI: 0.97–1.68, P = 0.08) (Fig 2). Further extracting fibrosis from late adverse effects in the subgroup analysis by specific adverse effect, we observed a significant association between rs861539 and fibrosis induced by radiotherapy (OR = 1.95, 95%CI: 1.01–3.75, *P* = 0.05) (Fig 4). For rs1799794, a significant association was observed with late adverse effect induced by radiotherapy (OR = 0.47, 95%CI: 0.26–0.86, *P* = 0.01) (Fig 2). For rs1799796, only one study was identified, and the statistical association was significant (OR = 3.98, 95%CI: 1.31–12.05, *P* = 0.01) (Fig 2).

#### Subgroup analysis by radiotherapy area

**Fig 5:**
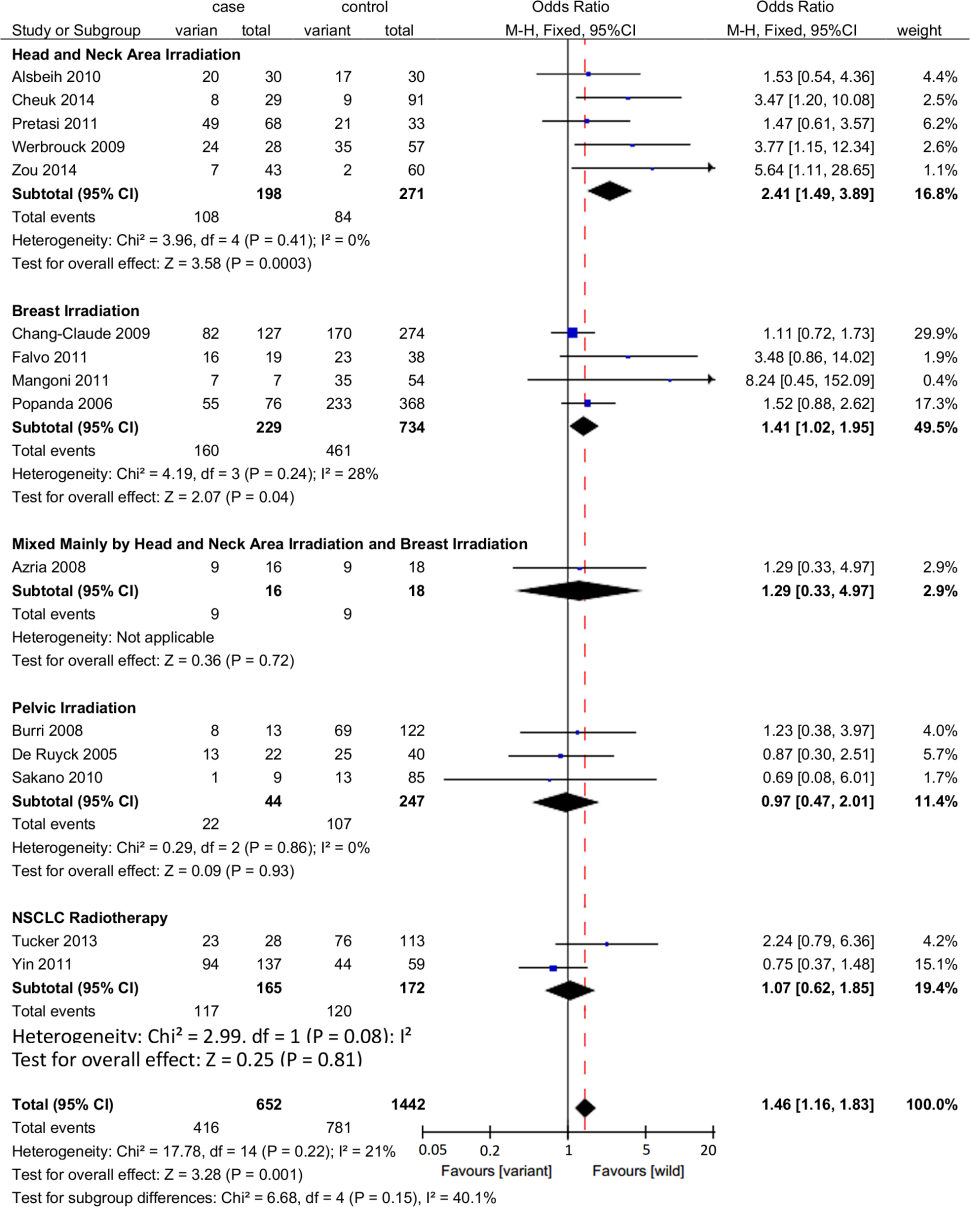
Forrest plot for the effect of rs861539 on adverse effects induced by radiotherapy of different body areas. A fixed-effects model was used. The square with the corresponding horizontal line represents the OR and 95%CI of each study. The area of the square reflects the weight of the study. The diamond represents the pooled OR and 95%CI.

Subgroup analysis was conducted by different radiotherapy area irrespective of the type of adverse effect. A significant association was identified between rs861539 and radiation-induced adverse effects of head and neck irradiation (OR = 2.41, 95%CI: 1.49–3.89, *P* = 0.0003) and breast irradiation (OR = 1.41, 95%CI: 1.02–1.95, *P* = 0.04), while no significant association was observed for lung irradiation (OR = 1.07, 95%CI: 0.62–1.85, *P* = 0.72) or pelvic irradiation (OR = 0.80, 95%CI: 0.38–1.68, *P* = 0.56) (Fig 5).

### Heterogeneity and sensitivity analyses

The heterogeneities between studies of all analyses were not significant except the analysis for the early effect of rs1799794 (*I*² = 89%, chi-squared *P* = 0.003). The pooled results were stable in the sensitivity analysis.

### Publication bias

**Fig 6:**
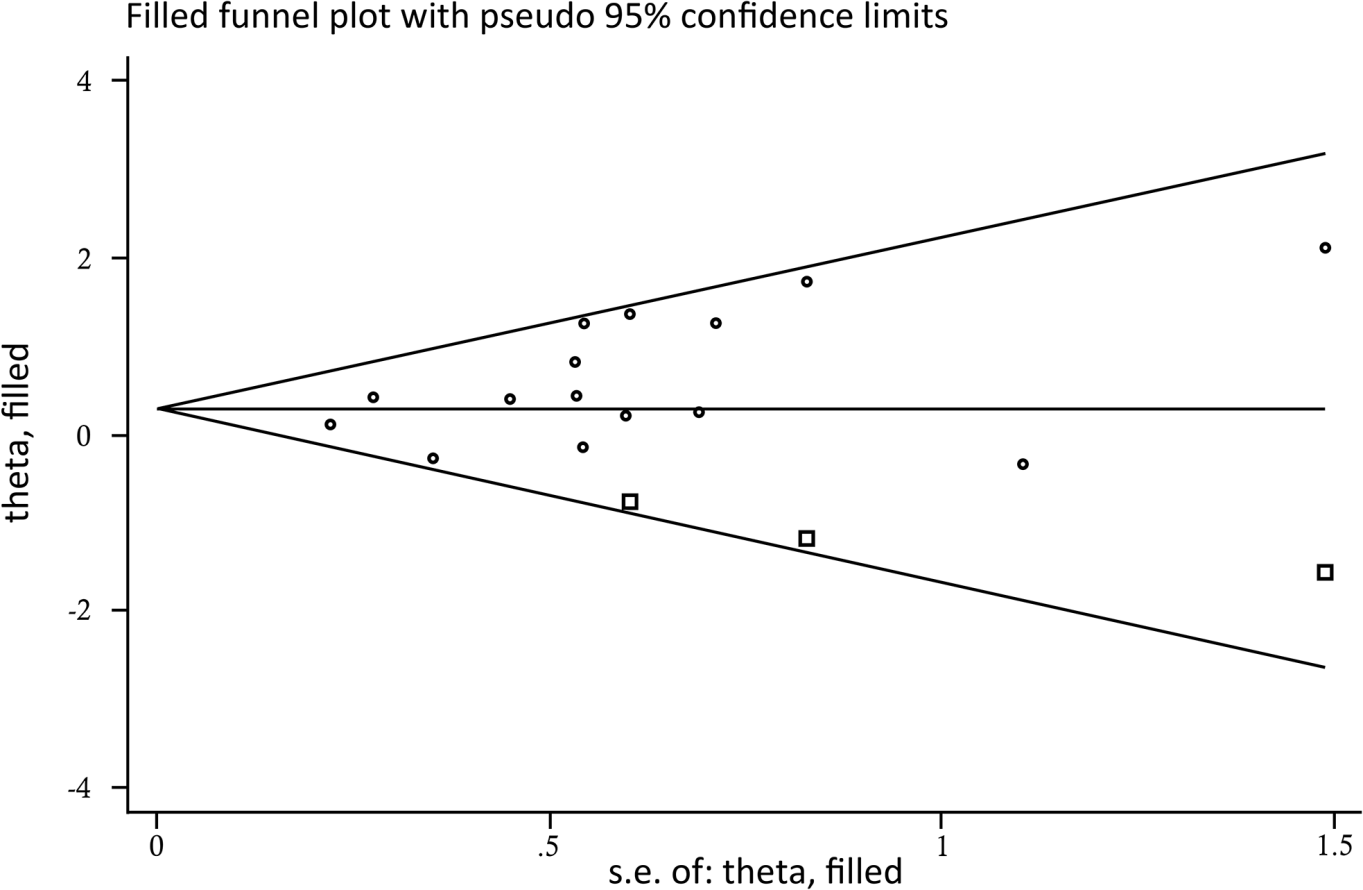
Begg’s funnel plot for the effect of rs861539 on radiation-induced adverse effects. Circles represent the actually included studies. Squares represent studies added using the “trim and fill” method.

The number of included studies for rs861539 was sufficient to evaluate the publication bias. The distribution of included studies in Begg’s funnel plot was visually symmetrical (Fig 6). However, the *p*-value of Egger’s test was 0.048, which indicates that potential publication bias may exist. Using the “trim and fill” method [29], three more potential studies were filled to reevaluate the pooled effect. The result was not altered significantly (pooled Est = 0.273, 95%CI: 0.049–0.496, *P* = 0.017) from the initial results (pooled Est = 0.354, 95%CI: 0.123–0.584, *P* = 0.003). The direction of the effect and the significant association were both constant, which indicates that the pooled result was stable and representative.

## Discussion

Radiation-induced adverse effects are commonly classified as early or late effects, depending on the time before manifestation of relevant clinical symptoms. Early effects occur during radiotherapy or within a few weeks after radiotherapy, while late effects emerge months to years after radiotherapy [2]. Early effects are often more serious in rapidly proliferating tissues, whereas late effects tend to occur in tissues with a slow turnover of cells [2,14]. Due to the inconformity effect of *XRCC3* on early and late effects previously reported and the possibility of different molecular mechanisms [44], we analyzed the early and late effects separately. The present meta-analysis systematically collected evidence linking *XRCC3* polymorphisms to the risk of adverse effects on normal tissue induced by radiotherapy. Three SNPs of *XRCC3* were included in our meta-analysis: *XRCC3* NM_005432.3:c.722C>T, NP_005423.1:p.Thr241Met (rs861539); *XRCC3* NM_005432.3:c.-316A>G (rs1799794); and *XRCC3* NM_005432.3:c.562-14A>G (rs1799796). *XRCC3* p.Thr241Met is the most commonly reported polymorphism of *XRCC3*. Our meta-analysis indicated that the *XRCC3* p.Thr241Met polymorphism is significantly associated with an elevated risk of radiation-induced early adverse effect.

A systematic review aimed to explore the association between 14 SNPs in 9 genes and radiotoxicity in normal tissues of the head and neck [45]. Of the seven included studies, three involved *XRCC3*. Due to obvious heterogeneity, no meta-analysis was undertaken. A positive association between SNPs in DNA repair genes and acute radiotoxicity events has been reported [45]. Since that report, two more articles evaluating the relationship between *XRCC3* polymorphisms and the risk of radiation-induced adverse effects in head and neck cancer (HNC) patients have been published, and they were included in the present meta-analysis. Our meta-analysis focused on *XRCC3* and provided more specific evidence. Subgroup analysis according to irradiated area revealed that rs861539 significantly correlates with an elevated risk of adverse effects induced by head and neck irradiation, with similar results observed for breast irradiation.

To date, six genome-wide association studies (GWASs) have been published on normal tissue radiobiology [46–51]. Most of the studies were performed using prostate cancer patient cohorts (one study used both prostate and breast cancer patients). *XRCC3* did not reach genome-wide significance in these studies. In the present meta-analysis, the pooled OR of pelvic irradiation was also not significant (OR = 0.97, 95%CI: 0.47–2.01, *P* = 0.81) (Fig 5), although only three studies involved pelvic irradiation.

For rs1799794, a significant association with late adverse effects was revealed (OR = 0.47, 95%CI: 0.26–0.86, *P* = 0.01), which indicates that rs1799794 polymorphism may have a protective effect against late adverse effects. Further studies are needed to confirm this conclusion. The analysis evaluating the effect of rs1799794 on early adverse effects yielded a significant heterogeneity, because the only two identified studies reached opposing conclusions. For rs1799796, only one study for each early and late effect was identified, and thus heterogeneity was not applicable. No definite conclusion can be made for rs1799794 and rs1799796, due to the relatively small number of identified studies.

*XRCC3* is an important protein in the process of homologous recombination, one of the two competitive mechanisms for repair of DNA DSBs [52–54]. Homologous recombination is generally considered high-fidelity [19]. By contrast, non-homologous end joining (NHEJ) is often error prone [19]. *XRCC3* p.Thr241Met is a non-conservative variant that may affect the structure of this DNA repair protein and lead to a deficiency in the homologous recombination pathway [55]. Consequently, the repair mechanism of DSBs could be shifted toward NHEJ, which promotes chromosome instability and further affects the cell’s ability to repair radiation injury [19]. However, GWASs of erectile dysfunction (ED) have revealed that the most significant SNPs lie in or near genes encoding biological activities involved in ED rather than DNA damage repair genes [47]. It should be noted that this conclusion was based on an endpoint that is a much more complex adverse effect compared with such adverse effects as skin toxicity or mucositis. Based on a similar hypothesis of influencing radiotoxicity, the association of *XRCC3* polymorphisms with cancer risk has also been extensively evaluated. Evidence from meta-analysis supports a positive association between the *XRCC3* p.Thr241Met polymorphism and the risk of bladder cancer [56–57], breast cancer [58–59], cervical cancer [60–61] and hepatocellular carcinoma [62–63]. However, in glioma [64–65], NSCLC [66–67], colorectal cancer [68–69] and gastric cancer [70–71] patients, the associations were not significant.

Although accumulating studies have evaluated the association between SNPs and radiation-induced adverse effects, no SNPs have been identified that can indicate which patients are at higher risk of normal tissue injury following radiotherapy [72]. Barnett et al. reevaluated 92 SNPs in 46 genes covering nearly all the SNPs previously reported to be associated with radiotherapy toxicity. A score system was developed to estimate the overall radiation toxicity rather than some specific adverse effect as was evaluated in the previous studies. None of the previously reported associations were confirmed using this method. The Q-Q plots indicated that no more significant associations than chance existed between the tested SNPs and overall late effects [73]. Nevertheless, a model that synthesizes multiple SNPs may possess greater power to predict normal tissue response in radiosensitive patients [40,74–76]. Azria et al. reported a higher risk of grade ≥3 toxicity in patients with ≥4 SNPs compared with those patients with <4 SNPs (OR = 9.3, 95%CI: 1.4–62, *P* = 0.003) [7]. Similarly, Sterpone et al. reported that patients with ≥3 SNPs had a higher risk of grade ≥2 toxicity than patients with <3 SNPs (OR = 2.42, 95%CI: 0.26–22.5, *P* = 0.39) [77]. However, these trials were not designed to evaluate the combined effect of multiple SNPs. After deliberately assessing the individual effects of selected SNPs, occasional combinations of involved SNPs were also performed. Further studies are needed to clarify the selection criterion for combined SNPs. The models developed from GWASs are more credible due to the sufficient genetic coverage. For example, one study presented a multivariate model comprising clinical factors and SNPs selected through a GWAS that achieved a sensitivity of 80% and a specificity of 70% in predicting ED following radiation therapy [47].

As a meta-analysis, heterogeneity among included studies should be considered. One of the most important potential sources of heterogeneity is the evaluation of miscellaneous cancer types together. However, the irradiated area, not the cancer type, is directly related to the occurrence of adverse effects induced by radiotherapy. Radiosensitivity varies according to anatomical site, but a common biological mechanism may occur in different irradiated organs. Hence, it is rational to include different cancer types when evaluating the adverse effects induced by radiotherapy on normal tissue [76]. Some of the heterogeneity among studies also derived from the heterogeneous treatment protocols, which was due to the characteristics of the radiotherapy and the inclusion of multiple cancer types in this meta-analysis. The radiotherapy parameters [5], including total dose, dose per fraction, field size, irradiation volume and depth of prescription point, were not identical. The majority of the treatment protocols in the included studies were based on multimodality treatment that is an important potential confounding factor aggravating the adverse effects, particularly when adjuvant or concurrent chemotherapy was involved. In addition, the criteria for assessing the adverse effects were not consistent, and the specific grades chosen to divide patients into case or control arm were also different among the included studies. Finally, 15 cohort studies and two case-control studies were included in our meta-analysis. Thus, differences in study design also contributed to the heterogeneity among studies.

In addition to heterogeneity, other limitations of the present meta-analysis should be noted when interpreting the results. First, publication bias may exist for rs861539; however, after adjustment using the “trim and fill” method [29], the result was stable in the direction of the effect, and still presented a significant association. Thus, we believe that the pooled result of rs861539 was not affected by any potential publication bias. Second, the eleven studies without sufficient data could not be expressed by weight in the pooled result, which may have generated some potential bias. Third, because miscellaneous irradiation areas and multiple adverse effects were evaluated in the present meta-analysis, subgroup analysis was conducted 3 times to reach more specific conclusions. The statistical power was reduced when the data were analyzed repeatedly. Forth, the sample sizes of some of the included studies and the numbers of studies in some of the subgroups were relative small, which also restricted the statistical power. In addition, our results were based on the raw data, which was unadjusted for certain confounding factors such as radiation dose or chemotherapy status.

To the best of our knowledge, the present meta-analysis is the first meta-analysis focusing on the association between *XRCC3* polymorphisms and the risk of radiation-induced adverse effects. In conclusion, the meta-analysis suggests that the *XRCC3* p.Thr241Met polymorphism is significantly associated with a higher risk of radiation-induced early adverse effects such as acute skin toxicity and mucositis. Although the association between rs861539 and late adverse effects was not significant, rs861539 was significantly correlated with a higher risk of fibrosis. In patients who received head and neck irradiation and breast irradiation, rs861539 was significantly correlated with a higher risk of adverse effects. Our results need to be further confirmed in well-designed large-scale clinical studies assessing the value of *XRCC3* polymorphisms in identifying patients at higher risk of radiation-induced adverse effect. These patients might benefit from individual radiotherapy regimens and early intervention for adverse effects accordingly.

## Supporting Information

S1 PRISMA Checklist PRISMA checklist. (DOC)

S1 Fig Forrest plot for the association between rs861539 and radiation-induced adverse effects by ethnicity. A fixed-effects model was used. The square with the corresponding horizontal line represents the OR and 95%CI of each study. The area of the square reflects the weight of the study. The diamond represents the pooled OR and 95%CI.
(TIF)

S1 Table Subgroup analysis of the association between rs861539 and radiation-induced adverse effects. *P*_Z-test_: *P* value of Z-test for overall effect. *P*_het_: *P* value of chi-squared based Q-test for heterogeneity. NA: not available.
(DOCX)

S2 Table Reason for exclusion of each article. (XLSX)

S3 Table Meta-analysis on genetic association studies checklist. (DOCX)
