# Supplementary figures and images for: Association between Single Nucleotide Polymorphisms in XRCC3 and Radiation-Induced Adverse Effects on Normal Tissue: A Meta-Analysis
Source: PLoS One. 2015 Jun 19;10(6):e0130388. doi: 10.1371/journal.pone.0130388 (PMC4474802; doi:10.1371/journal.pone.0130388)

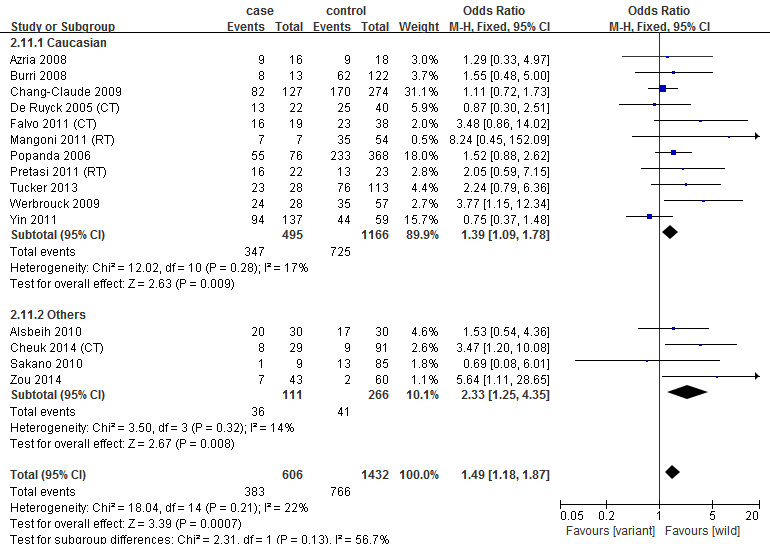

Supplement: S1 Fig — A fixed-effects model was used. The square with the corresponding horizontal line represents the OR and 95%CI of each study. The area of the square reflects the weight of the study. The diamond represents the pooled OR and 95%CI. (TIF) [file pone.0130388.s002.tif]
