# Supplementary material for: Association between Single Nucleotide Polymorphisms in XRCC3 and Radiation-Induced Adverse Effects on Normal Tissue: A Meta-Analysis
Source: PLoS One. 2015 Jun 19;10(6):e0130388. doi: 10.1371/journal.pone.0130388 (PMC4474802; doi:10.1371/journal.pone.0130388)
Supplement: S1 Table — P Z-test: P value of Z-test for overall effect. P het: P value of chi-squared based Q-test for heterogeneity. NA: not available. (DOCX) [file pone.0130388.s003.docx]

Table 2: subgroup analysis of the association between rs861539 and radiation induced adverse effect.

| **Subgroup** | **No. of studies** | **OR [95% CI]** ^a^ | ***P*_z-test_** | ***I*^2^** | ***P*_het_** |
| --- | --- | --- | --- | --- | --- |
| **Irradiation area** |  |  |  |  |  |
| Head and neck | 5 | 2.41 [1.49, 3.89] | 0.0003 | 0% | 0.41 |
| Breast | 4 | 1.41 [1.02, 1.95] | 0.04 | 28% | 0.24 |
| Pelvic | 3 | 0.97 [0.47, 2.01] | 0.93 | 0% | 0.86 |
| Lung | 2 | 1.07 [0.62, 1.85] | 0.81 | 67% | 0.08 |
| Other ^b^ | 1 | 1.29 [0.33, 4.97] | 0.36 | NA | NA |
| **Adverse effect** |  |  |  |  |  |
| Acute skin toxicity | 3 | 1.86 [1.13, 3.05] | 0.01 | 13% | 0.32 |
| Mucositis | 2 | 2.89 [1.24, 6.76] | 0.01 | 0% | 0.49 |
| Gastrointestinal morbidity | 1 | 0.69 [0.08, 6.01] | 0.74 | NA | NA |
| Fibrosis | 3 | 1.95 [1.01, 3.75] | 0.05 | 0% | 0.43 |
| Radiation pneumonitis | 2 | 1.07 [0.62, 1.85] | 0.81 | 67% | 0.08 |
| Other late effect | 4 | 1.16 [0.80, 1.68] | 0.43 | 32% | 0.22 |
| **Ethnicity** |  |  |  |  |  |
| Caucasian | 11 | 1.39 [1.09, 1.78] | 0.009 | 17% | 0.28 |
| Others | 4 | 2.33 [1.25, 4.35] | 0.008 | 14% | 0.32 |

***P*_Z-test_:** *P* value of Z-Test for overall effect.

***P*_het_:** *P* value of Chi² based Q-test for heterogeneity.

NA: not available.

^a^ Fixed-effect model used.

^b^ Mixed mainly by HNC and breast cancers.
